# Implementation of and experiences with new automation

**DOI:** 10.1155/S1463924600000353

**Published:** 2000

**Authors:** Ifte Mahmud, David Kim

**Affiliations:** Novartis Pharmaceuticals Corporation Su ern NY USA

## Abstract

In an environment where cost, timeliness, and quality drives the business, it is essential to look for answers in technology where these challenges can be met. In the Novartis Pharmaceutical Quality Assurance Department, automation and robotics have become just the tools to meet these challenges. Although automation is a relatively new concept in our department, we have fully embraced it within just a few years. As our company went through a merger, there was a significant reduction in the workforce within the Quality Assurance Department through voluntary and involuntary separations. However the workload remained constant or in some cases actually increased. So even with reduction in laboratory personnel, we were challenged internally and from the headquarters in Basle to improve productivity while maintaining integrity in quality testing. Benchmark studies indicated the Suffern site to be the choice manufacturing site above other facilities. This is attributed to the Suffern facility employees' commitment to reduce cycle time, improve efficiency, and maintain high level of regulatory compliance. One of the stronger contributing factors was automation technology in the laboratoriess, and this technology will continue to help the site's status in the future. The Automation Group was originally formed about 2 years ago to meet the demands of high quality assurance testing throughput needs and to bring our testing group up to standard with the industry. Automation began with only two people in the group and now we have three people who are the next generation automation scientists. Even with such a small staff,we have made great strides in laboratory automation as we have worked extensively with each piece of equipment brought in. The implementation process of each project was often difficult because the second generation automation group came from the laboratory and without much automation experience. However, with the involvement from the users at ‘get-go’, we were able to successfully bring in many automation technologies. Our first experience with automation was SFA/SDAS, and then Zymark TPWII followed by Zymark Multi-dose. The future of product testing lies in automation, and we shall continue to explore the possibilities of improving the testing methodologies so that the chemists will be less burdened with repetitive and mundane daily tasks and be more focused on bringing quality into our products.


Journal of Automated Methods & Management in Chemistry, Vol. 22, No. 6 (November–December 2000) pp. 199–202

### Implementation of and experiences with new automation

**Ifte Mahmud and David Kim**

*Novartis Pharmaceuticals Corporation, Su ern, NY, USA*

*In an environment where cost, timeliness, and quality drives the business, it is essential to look for answers in technology where these challenges can be met. In the Novartis Pharmaceutical Quality Assurance Department, automation and robotics have become just the tools to meet these challenges. Although automation is a relatively new concept in our department, we have fully embraced it within just a few years. As our company went through a merger, there was a significant reduction in the workforce within the Quality Assurance Department through voluntary and involuntary separations. However the workload remained constant or in some cases actually increased. So even with reduction in laboratory personnel, we were challenged internally and from the headquarters in Basle to improve productivity while maintaining integrity in quality testing. Benchmark studies indicated the Su ern site to be the choice manufacturing site above other facilities. This is attributed to the Su ern facility employees' commitment to reduce cycle time, improve efficiency, and maintain high level of regulatory compliance. One of the stronger contributing factors was automation technology in the laboratoriess, and this technology will continue to help the site's status in the future. The Automation Group was originally formed about 2 years ago to meet the demands of high quality assurance testing throughput needs and to bring our testing group up to standard with the industry. Automation began with only two people in the group and now we have three people who are the next generation automation scientists. Even with such a small sta , we have made great strides in laboratory automation as we have worked extensively with each piece of equipment brought in. The implementation process of each project was often difficult because the second generation automation group came from the laboratory and without much automation experience. However, with the involvement from the users at 'get–go', we were able to successfully bring in many automation technologies. Our first experience with automation was SFA/SDAS, and then Zymark TPWII followed by Zymark Multidose. The future of product testing lies in automation, and we shall continue to explore the possibilities of improving the testing methodologies so that the chemists will be less burdened with repetitive and mundane daily tasks and be more focused on bringing quality into our products.*

### Introduction

When we hear the word 'Automation', a mixture of emotions can be evoked, initially bringing fear. It is quite understandable that a person unexposed to any type of automation technology can dramatically fear the unknown—scenes flashing through one's mind from a movie of a robotic monster wreaking havoc upon mankind, thoughts of the starving family of a worker whose job is replaced by a robot that chugs along in an assembly line, and so on. However, as we come to our senses and begin to look around, we realize the significant impact of automation and how it has improved our lives. If we take the definition of 'automation' from Webster's dictionary, it simply means 'the technique of making an apparatus, a process, or a system operate automatically'. So in reality, we live in an age of automation, unable to imagine living without the benefits of coffee makers, washers and dryers, and VCRs.

When we recognize the truth about the positive influence of automation technology in our daily lives, the fear of laboratory automation can easily be dissolved and a sense of excitement takes over. The vision of automation bringing relief to chemists from mundane and repetitive tasks and increasing productivity and efficiency thereby contributing to the overall success of the company can now be a reality. This paper, though in many parts non-technical, is critical in the understanding of how we can bring new automation technology to an environment specific to quality assurance (QA) in pharmaceutical industries by breaking the 'invisible yet very real barrier' between fear and excitement. A great technology is only great to the degree it is understood and accepted by its users, so this paper will deal with challenges surrounding both people and related technical issues.

### History of automation at Novartis

Although the big push for automation in QA laboratories is recent, the term is certainly not new at Novartis. During the pre-merger era, many of the finished products in tablet form were tested for content uniformity with 'Technicon SolidPrep and Autoanalyzers'. It was somewhat of a predecessor to TPWII Workstation in that both systems utilized the homogenizer chamber—although the former lacked computer-generated operation. However, the difficulty with Technicon Autoanalyzers was in troubleshooting the numerous reagent lines and glass loops that introduced the required chemical reactions through a peristaltic pump system.

Another automation technology during that time was an on-line multibath dissolution unit for applications specific to patches, transdermal systems, and extended release tablets. The unit was a semi-automated system for dissolution profiles which pulled samples at designated time-points to a HP8452 spectrophotometer for reading, calculation, and generation of reports. The next generation of dissolution solution would follow much later with the Zymark Multidose System that completely automated dissolution from media dispensing all the way to report generation.

These two systems are just some examples of how automation began to play a significant role in the efficient processing of samples in the laboratories. Then the merger occurred between Ciba-Geigy and Sandoz, resulting in the formation of a new pharmaceutical entity called Novartis.

### Post-merger challenges

The merger and therefore the creation of Novartis resulted in challenges that had to be addressed almost immediately. These challenges included reducing the workforce, cutting costs, increasing productivity, transferring products from other sites, and benchmarking the Suffern site as the premier manufacturing site. As these challenges surfaced, the Quality Assurance Department (functioning under Technical Operations) responded quickly. One of the responses was the formation of an Automation Support Group to bring in systems that would increase efficiency and productivity and therefore drastically cut costs for the laboratory operations.

The greatest difficulty in bringing new automated systems into the laboratoriess was being able to sell the idea of automation benefits to the user community, who, by in large, had little interest or awareness about automation. This brought a negative 'domino' effect to the evaluation and the implementation of new automation technology. This was evident in the overall process of qualifying a content uniformity workstation from Source for Automation, Inc. The unit from Source for Automation, known as a Solid Dosage Assay System (SDAS), was met with resistance from the laboraty staff from the beginning. People were so used to doing everything manually that they showed a lack of support and cooperation when the system came into the laboratory. The result was a long and arduous implementation process. A similar path was observed for the implementation of the Zymark Multidose Automated Dissolution System.

### Overcoming the biggest challenge

Of all the challenges, the issues related to the user community, comprised primarily of laboratory bench chemists, were of greatest concern. The question was 'how could people be motivated to receive and utilize automation technology with enthusiasm?' It is very difficult when people resist changes and when their old habits just will not die. The key was to bring awareness and knowledge about automation to the laboratory people so the interest and the initiative would be from them. They needed to know that automation is about working smarter, increasing productivity and staying competitive, and freeing up time for other thinking tasks. To do this required an informational presentation. This was accomplished through a half-day seminar sponsored by Novartis and Zymark.

Laboratory management and representative chemists from each product group were invited and the seminar proved to be successful. The audience first received an overview of how automation can bring short-term and long-term benefits without necessarily reducing the headcount. Following the overview was a presentation by Zymark specific to their products so the audience could make a connection between theory and practice. The response after the seminar was tremendously positive. There were a lot of questions based on interest, and the participants began already to speak of possible automation needs and showed desire to be actively involved.

Following this event and initiation of several automation projects, a step was taken to establish an 'Automation Partnership' between the Automation Support Group and the QA testing groups. This is illustrated by a flow chart in figure 1 which shows the assignment of responsibilities. The chart displays the importance of each group's involvement in successfully implementing a system from evaluation to final implementation and training on the automated system. The highlighted box indicates an extensive involvement required from the user group in completing the studies necessary for specific method needs. '**QA**' identifies the testing group personnel whereas '**A**' identifies the automation group personnel.

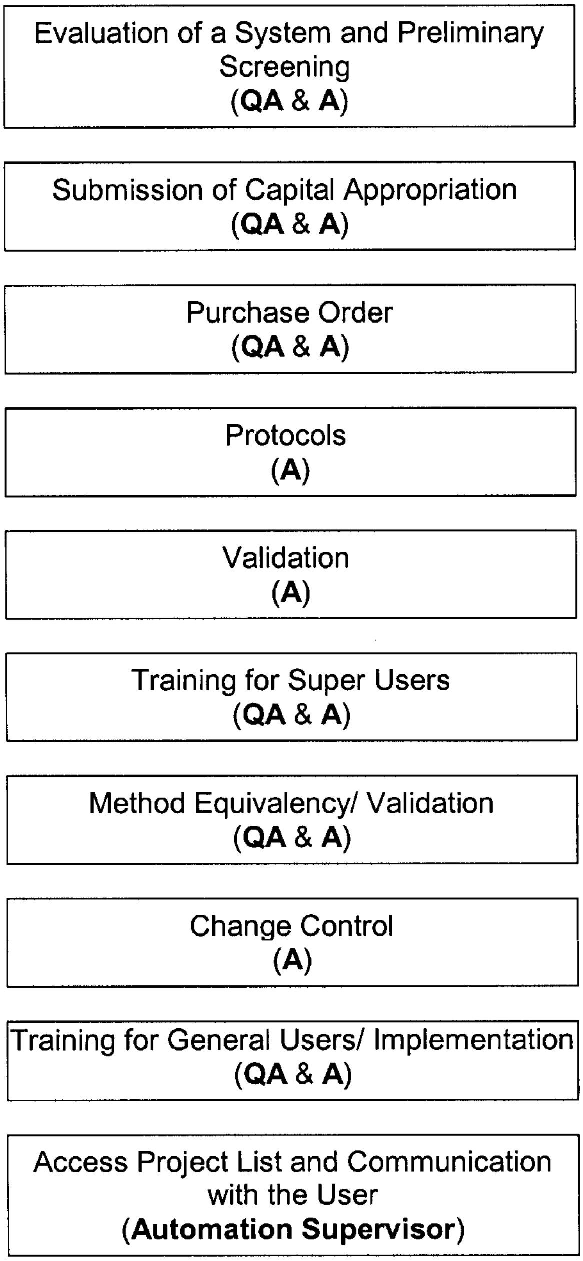

*Figure 1. Assignment of responsibilities.* ***QA****, testing group personnel;* ***A****, automation group personnel.*

As we look at this flow chart, we must emphasize that when a user from the testing group is involved from the initial phase of the project, he/she is more likely to take ownership of the instrument and participate not only in the implementation of the instrument but in its maintenance as well. When the users are motivated from the beginning, the rate of success in implementation increases drastically. As a user (a bench chemist) is invited to visit possible vendors and as the user has some say in the evaluation and the purchase of an automated system, there will be a higher level of commitment for utilization. Table 1 is an example of a chart that may be introduced to the users so different activities and expectations are defined clearly before the start of a project. This can be one of many ways to motivate and encourage the user in their involvement with automation projects.

### Personal experiences with automation implementation

When 1999 began, four different projects were already either in process or being initiated as a result of joint evaluation effort by the testing and the automation group. The decision came from management to apply automation to high volume products and to tests such as content uniformity and dissolution. Obstacles came from, first, the fact that the new year brought changes in the automation group with a complete turnover of the staff. Even though the automation team was already in place, the changeover in the members meant a new beginning, having to retrace the steps in the project to the first stage. At this point the commitment was already made to complete some of the projects on time and under budget. The projects started by the old members of the automation group had to be re-evaluated and user support brought in where required.

As projects progressed, we as automation support group members faced other barriers. Many of the problems dealt with system validation issues and execution of plans (outlined in table 2). These problems were systematically resolved as they surfaced and gave us a good picture of how upcoming projects can be anticipated and handled. Figure 2 shows a diagram of a 'Project Life Cycle' showing how everything begins with motivation and goes around, coming back to motivation. The diagram also shows how the success of each segment is crucial in the next step of the circle. We have adopted this approach because it is a step-by-step approach constantly fuelled by motivation. The motivation on the automation and the user team will keep the project moving even as we face on-going challenges.

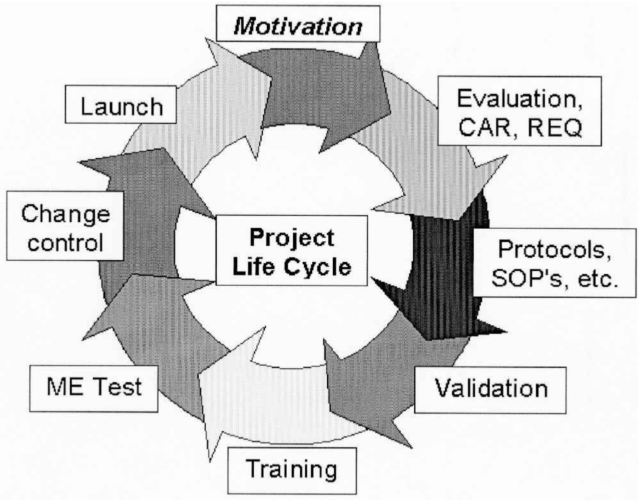

*Figure 2. Projected life cycle.*

*Table 1. Responsibility chart.*

| Tasks | Project Manager | Validation Group Lead | QC Group Lead | Super User-QC Scientist | Deadline |
|---|---|---|---|---|---|
| ME protocol | A | P | A | | |
| Shipment installation | | P | | | |
| Validation IQ, OQ, PQ | | P | | S | |
| Validation report | A | P | A | | |
| ME Testing | | S | | P | |
| ME reports | A | P | A | | |
| Change control | A | P | A | | |
| Training | | P | | S | |
| Launch | | | | | |

A, Approver; P, Primary Responsible Person; S, Secondary Responsible Person.

*Table 2. System validation issues and execution of plans.*

| System validation issues | Execution of plans |
|---|---|
| Vendor audit requirement as per Novartis Quality Module 5.2 | Calibration procedure requirement |
| Protocol approval by Computer System Validation Committee | SOP requirement to cover method equivalency testing |
| Stress test requirement | Change control documents |
| Function risk assessment | Operating procedure requirement |
| Y2K issues | General test requirement (GT) |

### Benefits of automation realized

As the automation projects were completed, everyone began to see the benefits gained through the implementation of these systems. The difference between the manual and the automated methods was evident with increases not only in efficiency but in quality as well. Looking at the examples of implementing specifically Zymark's TPWII Content Uniformity Workstation and Multidose Automated Dissolution System, the benefits are clear. With TPWII, there is no more tedious pipetting, saving time and glassware, especially for content uniformity testing. This gives chemists more time for validating reports and other thinking tasks thereby contributing to faster sample turnaround time. The automated method is superior to the manual method which is tedious, time consuming, wastes manpower and glassware, exposes harmful chemicals, and adds to testing errors.

Looking at the benefits of the Multidose Automated Dissolution System, one can easily see how the flexibility plus the efficiency of the system gives significant gains for the testing of products. Some of the observable advantages of the system include: rapid and accurate vessel filling with selected media, accurate temperature monitoring, single and multipoint dissolution, efficient and rapid vessel cleaning, up to 8 batches per setup, and interfacing capability to HPLC, UV/Vis, or a fraction collector. In addition, the automated systems bring cost savings that can be quantitatively measured. The projected savings for Zymark Multidose Plus can be seen in table 3. Table 4 shows additional examples of cost savings for a different type of automated dissolution system. Each table indicates a specific example of product testing.

### The future of automation at Novartis

In order to stay competitive in the pharmaceutical industry, it is necessary to pursue excellence in every possible facet of cost, timeliness, and quality. Automation of testing processes in the laboratory is a vital means of achieving success in those areas. So as we look to the future of automation at Novartis, our sights are on globalization of automation for Basle and various sites in US including East Hanover (NJ), Suffern (NY), Lincoln (NE), and Broomfield (CO). This would facilitate the transfer of method for a product. We would be able to send a method on a floppy disc or even electronically to the receiving site with minimal testing required.

As we press on with the future of automation in the QA environment, together as testing and automation groups, we shall be more proactive and not reactive in assessing the needs in the laboratory and in finding appropriate solutions—solutions that not only bring increases in productivity and cost savings but also bring the working relationships of the automation and the user groups to a whole new level. We can never underestimate the effort in automating a process in the QA laboratories; however, with proper planning and preparation supported by insight through prior technical knowledge, the future of automation is secure at Novartis.

### Trademarks

Source for Automation, Inc (SDAS)

Zymark (TPWII, Multidose, Multidose Plus)

Technicon (Solidprep, Autoanalyzers)

Hewlett Packard (HP8452)

Novartis Pharmaceuticals Corporation (Quality Module 5.2)

*Table 3. Projected savings on Diovan capsules (a transfer product) using Multidose Plus.*

| Activity | Yearly saving |
|---|---|
| Reduction in standard hours | $58 875.00 |
| Increased testing capacity | $25 200.00 |
| Use of the instrument for additional product | $22 125.00 |
| Total potential savings/year | $106 200.00 |

*Table 4. Projected savings on Tegretol XR tablets using Distek/HP8453 system.*

| Activity | Yearly Saving |
|---|---|
| Transfer two second shift analysts back to first shift | $16 500.00 |
| Increased testing capacity | $9 600.00 |
| Reduction in standard hours | $18 000.00 |
| Flexibility to begin test at any time of the day | $9 600.00 |
| Use of the instrument for additional product | $7 000.00 |
| Total potential savings/year | $60 700.00 |

